# Supplementary material for: Harnessing γ-TMT Genetic Variations and Haplotypes for Vitamin E Diversity in the Korean Rice Collection
Source: Antioxidants (Basel). 2024 Feb 14;13(2):234. doi: 10.3390/antiox13020234 (PMC10886147; doi:10.3390/antiox13020234)
Supplement: Supplementary file 1 [file antioxidants-13-00234-s001.zip › Supplementary Figures.pdf]

Supplementary Figures

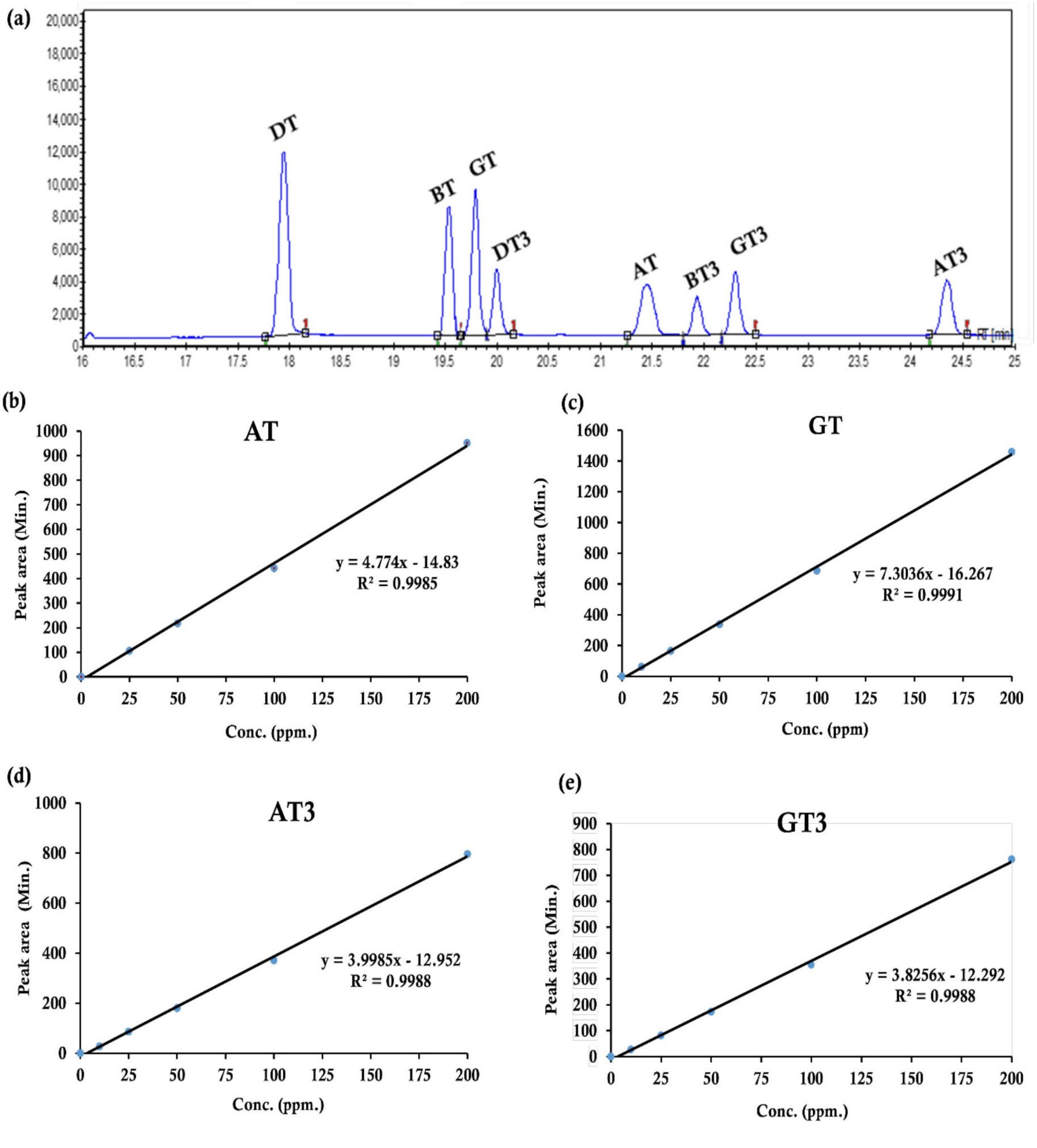

Figure S1. Vitamin E standard (**a**) Chromatogram of mixed standard for eight tocochromanol isomers; AT, α-tocopherol; BT, β-tocopherol; GT, γ-tocopherol; DT, δ-tocopherol; AT3, α-tocotrienol; BT3, β-tocotrienol; GT3, γ-tocotrienol; DT3, δ-tocotrienol (**b–e**) standard curve for vitamin E quantification in rice sample.

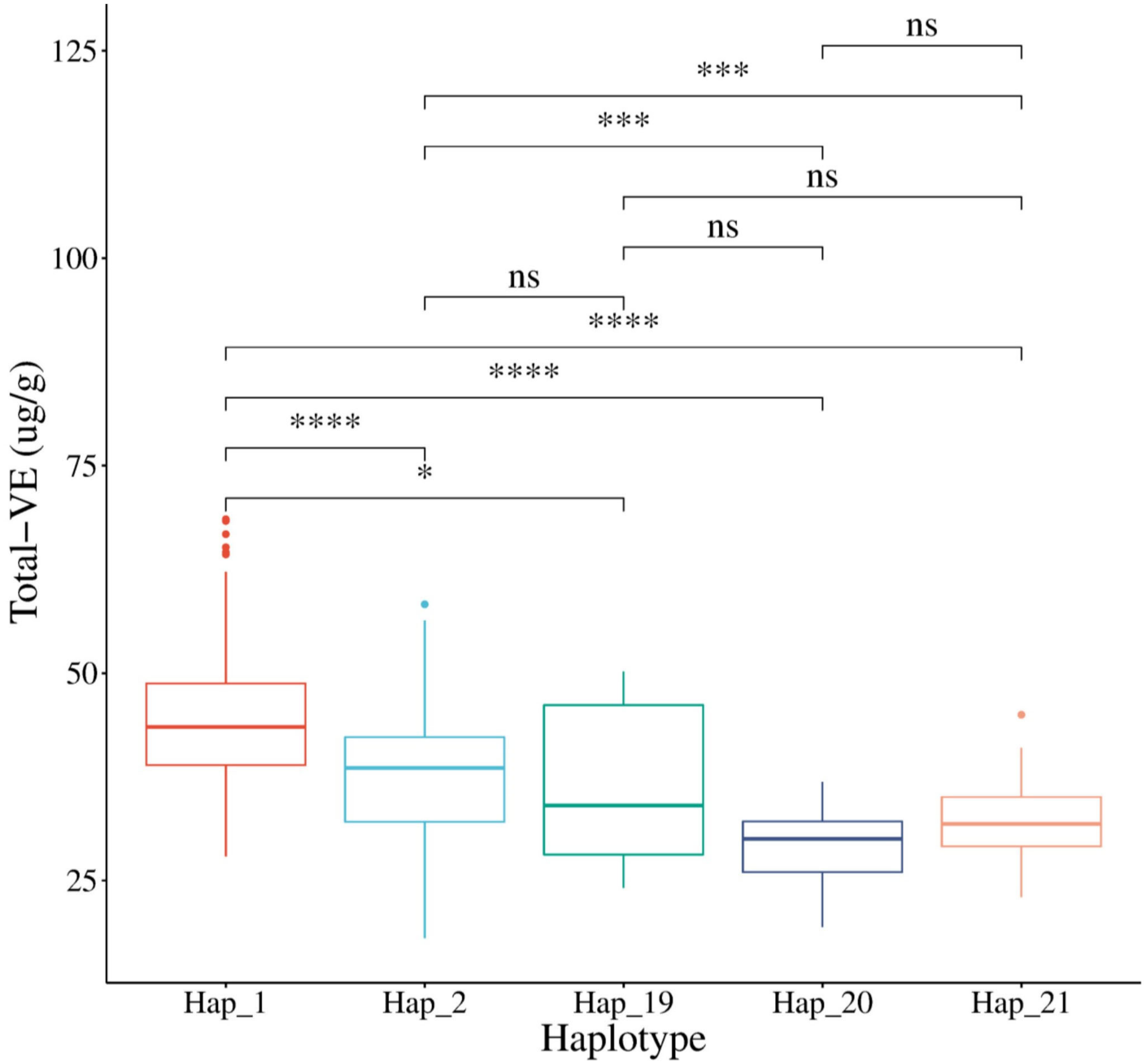

Figure S2. Variation in total vitamin E content among the haplotypes. The significant difference between each haplotype was investigated with $p < 0.0001$, 0.001, 0.05 indicating ****, ***, * based on t-test statistics.

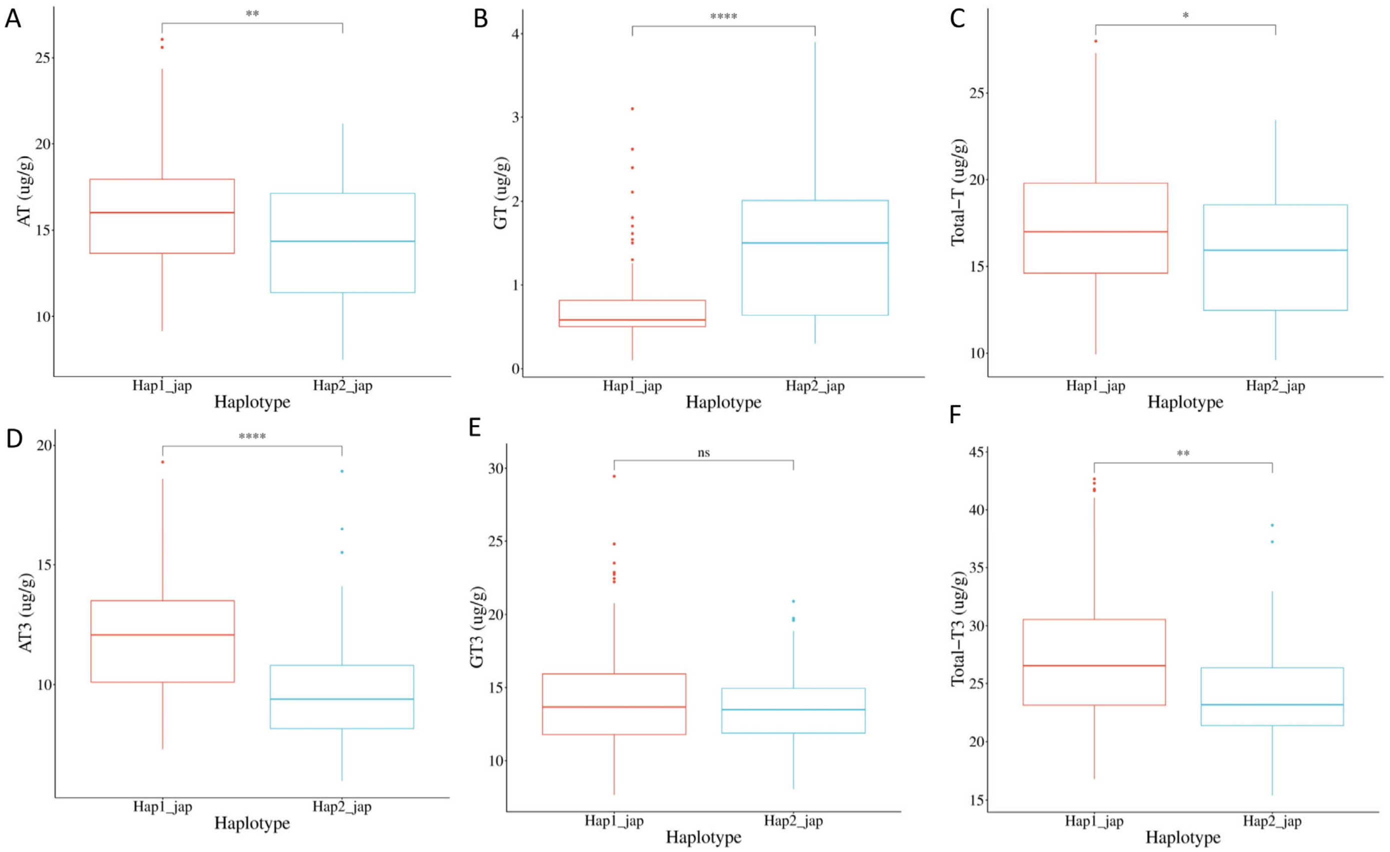

Figure S3. Evaluation of *japonica* haplotypes impact on vitamin E profile. Boxplot showing the impact of haplotypes on (**A**) AT, α-tocopherol; (**B**) GT, γ-tocopherol; (**C**) Total_T, total tocopherol; (**D**) AT3, α-tocotrienol; (**E**) GT3, γ-tocotrienol; (**F**) Total_T3, total tocotrienol. The significant difference between each haplotype was investigated with $p < 0.0001$, 0.01, 0.05 indicating ****, **, * based on t-test statistics.

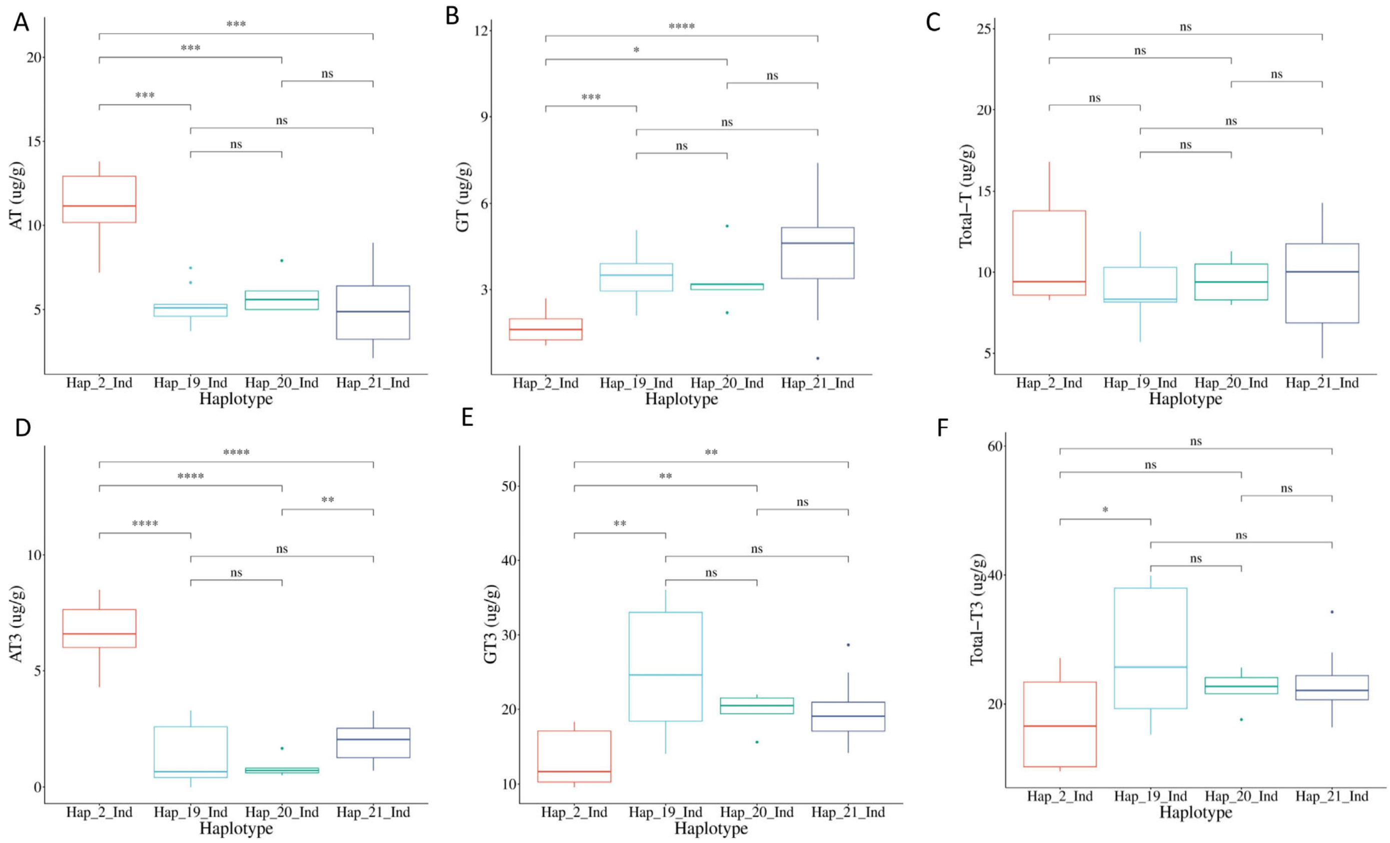

Figure S4. Evaluation of *indica* haplotypes impact on vitamin E profile. Boxplot showing the impact of haplotypes on (**A**) AT, α-tocopherol; (**B**) GT, γ-tocopherol; (**C**) Total_T, total tocopherol; (**D**) AT3, α-tocotrienol; (**E**) GT3, γ-tocotrienol; (**F**) Total_T3, total tocotrienol. The significant difference between each haplotype was investigated with $p < 0.0001$, 0.001, 0.01, 0.05 indicating ****, ***, **, * based on t-test statistics.

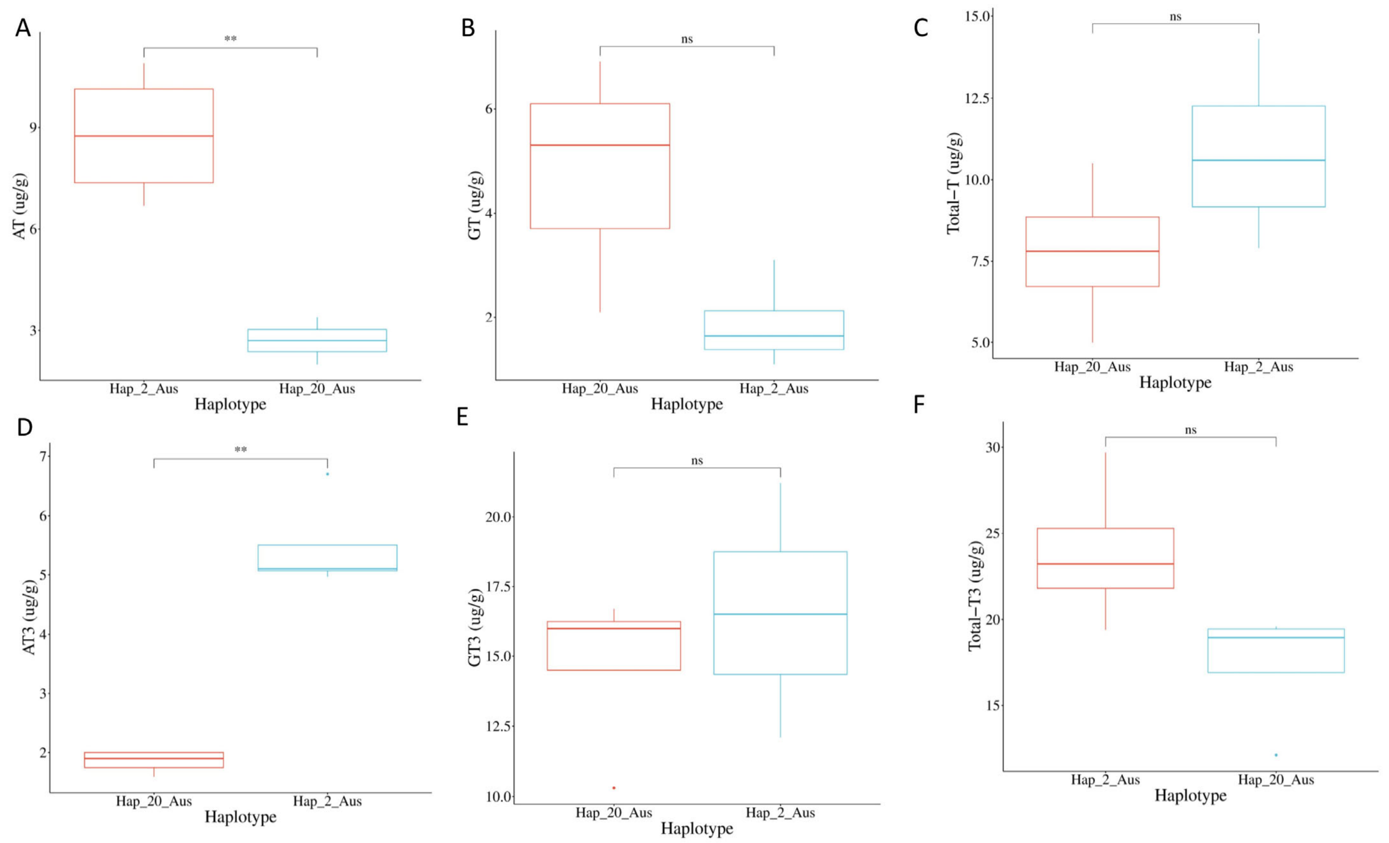

Figure S5. Evaluation of *aus* haplotypes impact on vitamin E profile. Boxplot showing the impact of haplotypes on (**A**) AT, α-tocopherol; (**B**) GT, γ-tocopherol; (**C**) Total_T, total tocopherol; (**D**) AT3, α-tocotrienol; (**E**) GT3, γ-tocotrienol; (**F**) Total_T3, total tocotrienol. The significant difference between each haplotype was investigated with $p < 0.01$ indicating ** based on t-test statistics.

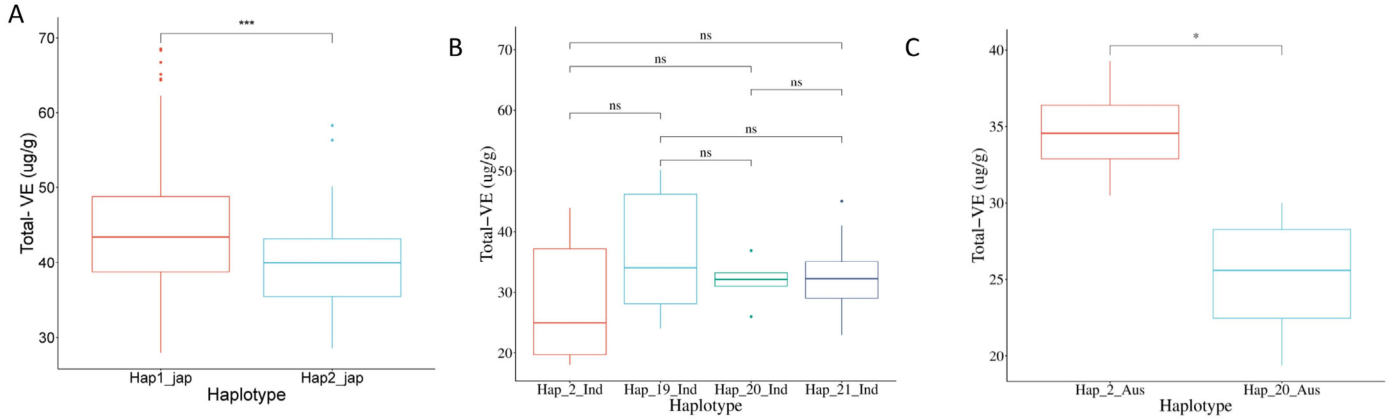

Figure S6. Evaluation of haplotypes impact on total vitamin E content. Boxplot showing the impact of haplotypes in (**A**) Japonica; (**B**) Indica; (**C**) Aus. The significant difference between each haplotype was investigated with $p < 0.001$, 0.05 indicating ***, * based on t-test statistics.
